# Engineered ZnO and CuO Nanoparticles Ameliorate Morphological and Biochemical Response in Tissue Culture Regenerants of Candyleaf (*Stevia rebaudiana*)

**DOI:** 10.3390/molecules25061356

**Published:** 2020-03-17

**Authors:** Muhammad Arslan Ahmad, Rabia Javed, Muhammad Adeel, Muhammad Rizwan, Qiang Ao, Yuesuo Yang

**Affiliations:** 1 Key Lab of Eco-restoration of Regional Contaminated Environment (Shenyang University), Ministry of Education, Shenyang 110044, China; arslan.slu@gmail.com; 2 Department of Tissue Engineering, China Medical University, Shenyang 110122, China; aoqiang@mail.tsinghua.edu.cn; 3 Beijing Key Laboratory of Farmland Soil Pollution Prevention and Remediation, College of Resources and Environmental Sciences, China Agricultural University, Beijing 100193, China; chadeel969@gmail.com; 4 Microelement research center, College of Resources and Environment, Huazhong Agricultural University, Wuhan 430070, China; m.rizwan110@hotmail.com; 5 Key Lab of Groundwater and Environment (Jilin University), Ministry of Education, Changchun 130021, China

**Keywords:** tissue culture, ZnO and CuO ENPs, Candyleaf, secondary metabolites, phytotoxicity

## Abstract

Sustainable production of secondary metabolites in medicinal plants by artificial culturing on the industrial scale has gained worldwide importance. Engineered nanoparticles (ENPs) play a pivotal role in the elicitation of compounds of medicinal value. This investigation explores the influence of ZnO and CuO ENPs on in vitro roots formation, non-enzymatic antioxidant activities, and production of steviol glycosides (SGs) in regenerants of Candyleaf, *Stevia rebaudiana*. ENPs were applied in 0, 2, 20, 200, and 2000 mg/L of concentration in the MS medium containing plant shoots. The percentage of rooting induced was 91% and 94% by applying ZnO ENPs (2 mg/L) and CuO ENPs (20 mg/L), respectively. Moreover, at 2 mg/L of ZnO and 20 mg/L of CuO ENPs, the high performance liquid chromatography studies determined the significantly greatest content of SGs; rebaudioside A (4.42 and 4.44) and stevioside (1.28 and 1.96). Phytochemical studies including total flavonoid content, total phenolic content, and 2,2-diphenyl-1-picryl hydrazyl-free radical scavenging activity were calculated highest by the regenerants grown in 2 mg/L of ZnO and 20 mg/L of CuO ENPs dosage. Both ZnO and CuO ENPs at 200 mg/L and 2000 mg/L of concentration induced adverse effects on plant biomass, antioxidant activities, and SGs content up to 1.22 and 1.77 for rebaudioside A and 0.21 and 0.25 for stevioside. Hence, the biochemical and morphophysiological responses of Candyleaf were elicited as a defense against ZnO and CuO ENPs applied under threshold limit. This artificial biotechnological technique holds great promise for continued production of natural antioxidants on commercial scale and our study has further strengthened this impact.

## 1. Introduction

Nanotechnology is revolutionizing in different research areas for the last decade [1] and engineered nanoparticles (ENPs) have found broader applications in electronics, agriculture, ceramics, medicine, etc. due to their extensive potentiality and versatility [2]. However, nanopesticide and nanofertilizer formulations, and drug delivery during plant transformation are best examples in the context of the role of ENPs in agriculture [3]. Effect of metallic and metallic oxide nanoparticles such as Ag, Au, Fe, MgO, and TiO_2_ on different crop plants like mung bean (*Phaseolus radiatus*), radish (*Raphanus sativus*), Cucumber (*Cucumis sativus*), corn (*Zea mays*), Asian rice (*Oryza sativa*), etc. has been studied [4,5,6,7,8]. Furthermore, the green synthesis of various nanoparticles has been rapidly progressing using leaf extracts of medicinal plants including purple fruited pea eggplant (*Solanuum trilobatum*), little ruby (*Alternanthera dentate*), Indian copperleaf (*Acalypha indica*), Malabar neem (*Melia dubia*), true aloe (*Aloe vera*), holy basil (*Ocimum sanctum*), and so on [9,10,11].

Production of ZnO and CuO ENPs is increasing worldwide and has reached to 550 tons/year in the case of ZnO ENPs [12] and 200 tons/year for CuO ENPs [13]. There are only a few studies exploring the effect of ZnO and CuO ENPs on different medicinal plants. ZnO ENPs (500–1500 mg/L) produce deleterious effects on seed germination and seedling growth of black mustard (*Brassica nigra*) [14] and 26.4% reduction in biomass has been observed in the case of *Fagopyrum esculentum* exposed to ZnO ENPs (10–2000 mg/L) [15]. Whereas, exposure of CuO ENPs (2000 and 4000 mg/L) has shown the decrease in root growth for buckwheat (*Fagopyrum esculentum*) [16]. Narendhran et al. [17] evaluated a less toxic effect of ZnO ENPs as compared to bulk ZnO on the seed germination of sesame (*Sesamum indicum*). Samart et al. [18] explored the influence of ZnO ENPs on photosynthetic pigment contents and antioxidant enzyme activities of non-photoperiod sensitive Asian rice (*Oryza sativa*) cultivars, and determined that there was no significant difference in the contents of photosynthetic pigments and a protective effect of antioxidant enzymes was evident by their accumulation in plant cells. Wang et al. [19] observed significant inhibition of root and shoot growth, and reduction in photosynthetic efficiency in tomato (*Solanum lycopersicum*) under ZnO ENPs exposure. Similarly, Margenot et al. [20] reported adverse effects of CuO ENPs on root physiology of lettuce (*Lactuca sativa*) and carrot (*Daucus carota*) crops.

Candyleaf (Family: Asteraceae) is the common name of *Stevia rebaudiana* due to its marvelous sweetness and flavor-enhancing property. It is a perennial herb indigenous to Brazil and Paraguay, and also abundantly occurs in China, Korea, and Thailand [21]. The major calorie-free steviol glycosides (SGs), i.e., rebaudioside A, stevioside, and rebaudioside C obtained from Candyleaf provide relief to diabetic, cancerous, and obese people [22,23]. It also covers many other remarkable medicinal purposes according to World Health Organization (WHO) findings [24]. Since changing climate adversely affects yield and metabolic content of Candyleaf in the field, many tissue culture protocols containing biotic/abiotic stressors have been optimized producing enhanced quantity of SGs under controlled laboratory conditions. The studies involving abiotic stress induced by chilling due to H_2_O_2_, salt (NaCl) stress, and plant growth regulators (PGRs) stress to Candyleaf have magnified the industrial importance of the plant tissue culture technique [25,26,27,28].

Bayraktar et al. [29] illustrated stevioside production by this biotechnological approach using elicitors including methyl jasmonate (MeJA), salicylic acid (SA), and chitosan (CHI). Similarly, Kazmi et al. [30] explored the growth induction, SGs production, and phytochemicals formation in in vitro adventitious roots grown under the presence of methyl jasmonate (MeJA), phenyl acetic acid (PAA), and melatonin elicitors. Moreover, SGs biosynthetic pathway on elicitation by different stressors has been discovered recently [31]. The escalation of bioactive pharmaceutical compounds in endangered and valuable medicinal plants under the influence of ENPs elicitation has recently become an active area of research. Different studies have been reported in a tissue culture grown Candyleaf in this regard. These studies encompass capped and uncapped metallic/metallic oxide nanoparticles like Fe, Cu, Si, Mg, Ag, and TiO_2_ affecting the growth dynamics and secondary metabolites of shoots and the callus of the Candyleaf [32,33,34,35,36,37,38,39]. The effect of ENPs on Candyleaf is studied in order to reveal the quantity and consequences of oxidative/abiotic stress caused by different ENPs concentrations, and to explore nanotoxicity mechanisms in this plant [40].

The present study involves the employment of ZnO and CuO ENPs to the regenerants of Candyleaf (*S. rebaudiana*). The effect of ZnO and CuO ENPs stress has previously been documented only in the shoots of Candyleaf grown by tissue culture [34,35]. However, the impact that both these ENPs create on in vitro rooting of this highly valuable medicinal plant has not been exploited so far. Our prime objective to perform this study is to reveal the influence of engineered ZnO and CuO ENPs causing elicitation during root formation and measurement of feasible secondary metabolites production in leaves of the Candyleaf. This research is anticipated to enhance our existing knowledge about the possible effects of ENPs on the medicinal component of produce.

## 2. Results

### 2.1. Characterization of ENPs

**Figure 1:**
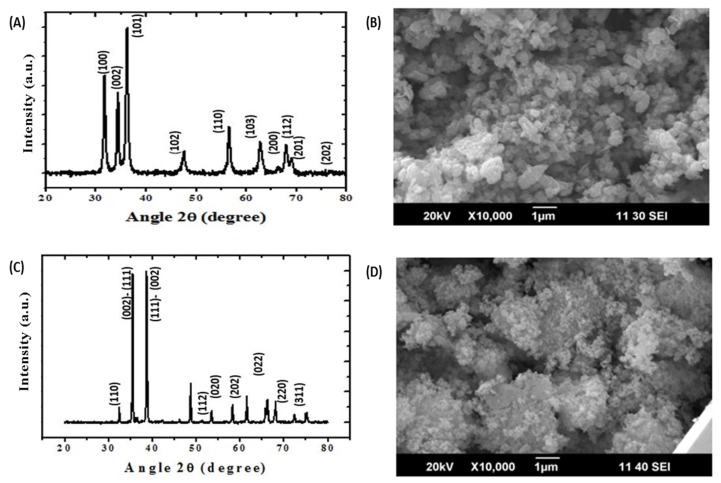
(**A**) X-ray diffractogram (XRD) of ZnO engineered nanoparticles (ENPs), (**B**) scanning electron micrograph (SEM) of ZnO ENPs, (**C**) X-ray diffractogram (XRD) of CuO ENPs, and (**D**) scanning electron micrograph (SEM) of CuO ENPs.

**Figure 2:**
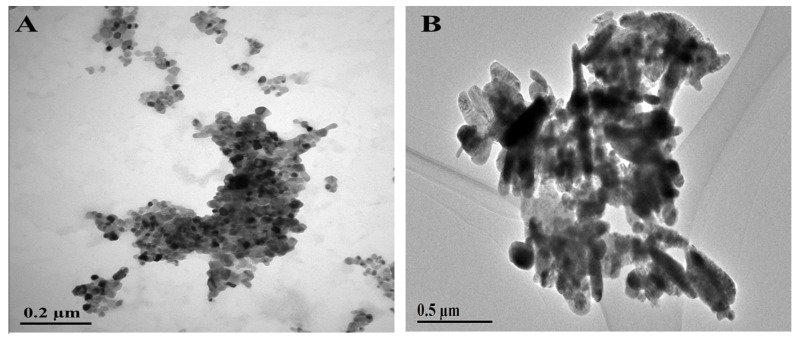
TEM image of (**A**) ZnO ENPs and (**B**) CuO ENPs.

The crystalline nature of ZnO ENPs as evident with the sharp peaks obtained with the powder XRD technique confirmed the hexagonal structure (Figure 1A). The peaks with high intensities reveal the purity of ZnO ENPs. The size of ZnO ENPs was calculated to be 26 nm using Scherrer’s formula [41]. Morphology of ZnO ENPs was studied using SEM. SEM micrograph shows the ZnO ENPs of hexagonal shape (Figure 1B). Figure 2A shows the TEM micrograph of ZnO ENPs. The average size of the ZnO ENPs was in the range of 20–30 nm. ZnO ENPs show the average zeta potential value of −12.8 mV (Table 1), which shows its incipient stability [42].

Crystal structure of CuO ENPs was studied using powder XRD analysis. The peaks in XRD spectrum reveal the monoclinic structure for CuO ENPs (Figure 1C). The size of CuO ENPs was calculated to be 27 nm using Scherrer’s formula [41]. The SEM observation shows the presence of CuO ENPs having monoclinical symmetry (Figure 1D). Figure 2B shows the TEM micrograph of CuO ENPs. The average size of the CuO ENPs was in the range of 25–30 nm. CuO ENPs shows the average zeta potential value of −11.7 mV (Table 1), which shows its certain level of stability [42].

### 2.2. Effects of ENPs on Plant Physiology

The results of (Table 2) show different morphological parameters during rooting of the Candyleaf under the elicitation of ZnO ENPs. The percentage of rooting (90.5%), mean length of regenerants (27.3 cm), mean length of roots (7.16 cm), mean number of roots (17.3), mean number of nodes (9.33), mean number of leaves (20.6), fresh weight of regenerants (0.80 g), and fresh weight of leaves (0.36 g) was obtained significantly highest under 2 mg/L of ZnO ENPs stress. All parameters were obtained significantly lowest when the stress of ZnO ENPs was 2000 mg/L. The overall trend was the increase of all physiology parameters at 2 mg/L threshold. Then, a sudden decline was observed by increasing concentration of ZnO ENPs from 20 to 2000 mg/L.

Table 3 reveals the comparison of morphological parameters of the Candyleaf plant raised by tissue culture under CuO ENPs elicitation. The percentage of rooting (94.2%), mean length of regenerants (21.1 cm), mean length of roots (8.54 cm), mean number of roots (26.3), mean number of nodes (11.06), mean number of leaves (19.3), fresh weight of regenerants (0.76 g), and fresh weight of leaves (0.36 g) were obtained significantly highest under 20 mg/L of CuO ENPs stress. Similar to the trend observed in the case of ZnO ENPs, here also regenerants demonstrated a rise of all physiological parameters until a certain threshold value, i.e., 20 mg/L in the case of CuO ENPs and became phytotoxic after that. Maximum phytotoxicity was observed at 2000 mg/L of CuO ENPs.

### 2.3. Evaluation of Steviol Glycosides

**Figure 3:**
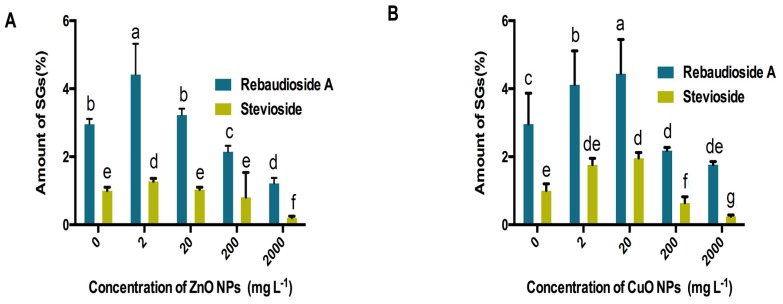
(**A**) Effect of ZnO ENPs and (**B**) effect of CuO ENPs at different concentrations ranging between 0 and 2000 mg/L on rebaudioside A content represented with blue bars and stevioside content indicated with yellow bars. Different letters indicate differences according to the Duncan’s multiple range test (*p* < 0.05).

Candyleaf regenerants produce the highest amount of steviol glycosides (rebaudioside A and stevioside) under the stress of ZnO ENPs at 2 mg/L, afterwards an increase in the concentration of ZnO ENPs progressively reduces the production of steviol glycosides. The amount of rebaudioside A increased from 2.96% in the control group up to 4.42% in treatment supplemented with 2 mg/L of ZnO ENPs and thereafter gradually decreased to 1.22% at 2000 mg/L of ZnO ENPs. Furthermore, stevioside content in regenerants of Candyleaf (*S. rebaudiana*) increased from 1.01% in the control group to 1.28% at 2 mg/L of ZnO ENPs followed by a gradual decrease in its production to 0.21% at 2000 mg/L of ZnO ENPs stress (Figure 3A).

Production of steviol glycosides (rebaudioside A and stevioside) in Candyleaf regenerants increased until a certain threshold value, i.e., 20 mg/L in the case of CuO ENPs, afterwards an increase in the concentration of CuO ENPs gradually diminished the production of steviol glycosides. The amount of rebaudioside A increased from 2.96% in the control group up to 4.44% in treatment supplemented with 20 mg/L of CuO ENPs and thereafter gradually decreased to 1.77% at 2000 mg/L of CuO ENPs. Furthermore, stevioside content in regenerants of Candyleaf increased from 1.01% in the control group to 1.96% at 20 mg/L of CuO ENPs followed by the gradual decrease in its production to 0.25% at 2000 mg/L of CuO ENPs stress (Figure 3B).

### 2.4. Estimation of Antioxidant Activities

**Figure 4:**
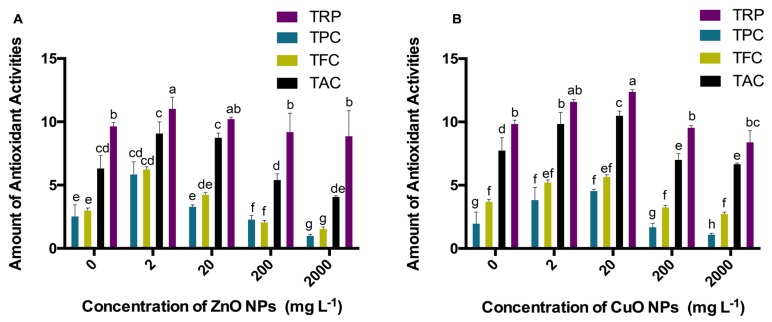
(**A**) Effect of ZnO ENPs and (**B**) effect of CuO ENPs at different concentrations ranging between 0 and 2000 mg/L on different antioxidant activities. Different letters indicate differences according to the Duncan’s multiple range test (*p* < 0.05).

**Figure 5:**
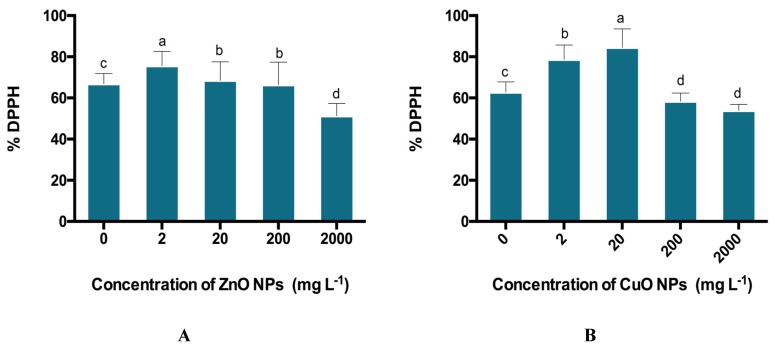
(**A**) Effect of ZnO ENPs and (**B**) effect of CuO ENPs at different concentrations ranging between 0 and 2000 mg/L on % DPPH inhibition activity. Different letters indicate differences according to the Duncan’s multiple range test (*p* < 0.05).

Total phenolic content (TPC), total flavonoid content (TFC), total antioxidant capacity (TAC), total reducing power (TRP), and DPPH free radical scavenging activity were calculated in Candyleaf regenerants under elicitation of ZnO and CuO ENPs. The highest amount of TPC (5.86 μg GAE/mg), TFC (6.25 μg QE/mg), TAC (9.09 μg AAE/mg), TRP (11.04 μg AAE/mg), and % DPPH inhibition (75.6%) was obtained at 2 mg/L of ZnO ENPs (Figure 4A and Figure 5A).

Under the elicitation of CuO ENPs, the highest amount of TPC (4.55 μg GAE/mg), TFC (5.67 μg QE/mg), TAC (10.05 μg AAE/mg), TRP (12.04 μg AAE/mg), and % DPPH inhibition (84.5%) was obtained at 20 mg/L (Figure 4B and Figure 5B). Moreover, the significantly lowest content of all antioxidant activities was shown by regenerants elicited under 2000 mg/L of both ZnO and CuO ENPs stress.

## 3. Discussion

Employment of ENPs in the tissue/cell culture industry of medicinal plants is limited despite a lot of progress having been made in the last decade in the agricultural nanotechnology sector. The available literature suggests that ENPs evoke abiotic stress in tissue/cell culture raised medicinal plants [43,44,45,46,47,48]. These studies illustrate the effects induced by ENPs on the growth and secondary metabolism of plants of medicinal significance. In this study, ZnO and CuO ENPs pose an oxidative stress to micropropagated roots of Candyleaf (*S. rebaudiana*), which have been revealed to tolerate this stress for up to 2 mg/L of ZnO and 20 mg/L of CuO ENPs. Previously, the influence of these ENPs on the in vitro grown shoots of Candyleaf has been documented. Those findings coincide with the current results where maximum threshold was achieved at 1 mg/L and 10 mg/L dosages of ZnO and CuO ENPs, respectively [34,35,36]. It is comprehended that an almost equal ENPs (ZnO and CuO) stress has been tolerated by the Candyleaf shoot and root organs. Hence, a promising content of secondary metabolites was achieved by the potent regenerants of Candyleaf in our investigation. Zafar et al. [49] also demonstrated the positive effect of ZnO ENPs (10 mg/L) on seedlings and stem explants of black mustard (*Brassica nigra*) with respect to various non-enzymatic antioxidant activities. However, Thunugunta et al. [50] reported negative influence of ZnO ENPs on the growth of eggplant (*Solanum melongena*). Regarding CuO ENPs exposure, a rise in polyphenolic content and other antioxidants was obtained by Singh et al. [51] in poison gooseberry (*Withania somnifera*). Despite of a few available studies, more investigation is required for comprehensive exploitation of the elicitation caused by ZnO and CuO ENPs in various medicinal plants grown in vitro.

**Figure 6:**
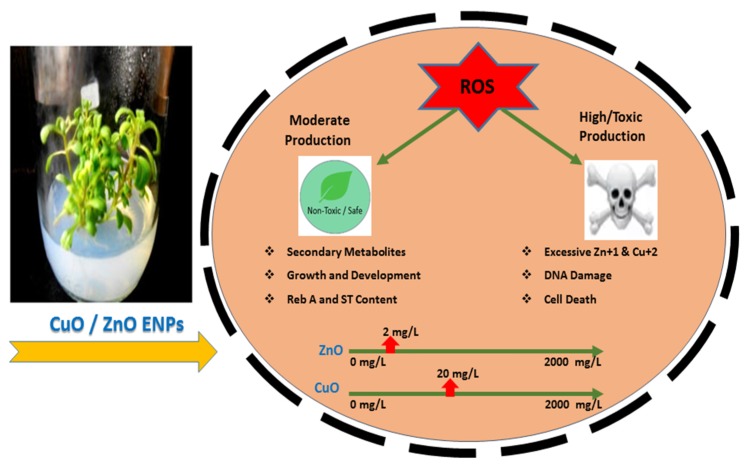
Mechanism illustrating the action of different dosages of ZnO and CuO ENPs for the induction of abiotic stress and stimulation of morphophysiological and biochemical responses in tissue culture regenerants of the Candyleaf due to the production of reactive oxygen species (ROS) in its cellular compartments.

The mechanism behind an increase of growth, SGs, and phytochemical antioxidant activities by the increasing concentration/dosage of ENPs and then, the immediate decline in the growth and metabolism after reaching a certain threshold might involve the abundance of reactive oxygen species (ROS) and free radicals of Zn^+1^ and Cu^+2^ ions (Figure 6). The ROS/toxic free radicals produced by ZnO and CuO ENPs are internalized into the plant cell wall, cell membrane, cytoplasm, and eventually nucleus. The translocation of ROS into the plant cells cause their ultimate destruction that occurs by the degradation of genomic DNA and mitochondrial membranes [52,53,54]. Moreover, the denaturation of proteins and peroxidation of lipids take place leading to mutagenesis. Consequently, the inflammatory signaling cascades are activated, and transcription of carcinogenic genes is stimulated causing genotoxicity. Apart from cellular responses, the size, surface, and composition of ENPs also cause oxidative stress [55]. It is the function of naturally synthesized antioxidants of the Candyleaf to fight with oxidative bullets but they cannot scavenge free radicals to that extent at higher concentration. Hence, the growth parameters and secondary metabolites show a fall after reaching the threshold barrier.

The maximum amount of phytotoxicity was obtained at 2000 mg/L in the current study by which physiological parameters, SGs, and non-enzymatic antioxidant activities mitigate to a minimum. The supreme toxicity of ZnO ENPs at 1000 mg/L of concentration was elucidated in corn (*Zea mays*) and cucumber (*Cucumis sativus*) by Zhang et al. [56]. Similarly, Zafar et al. [14] also endorsed the toxicological phenomena at 1000 mg/L in black mustard (*Brassica nigra*). Furthermore, the studies of Yang et al. [6] on crop plants, corn (*Zea mays*) and Asian rice (*Oryza sativa*), reveal CuO ENPs to be more phytotoxic than ZnO ENPs. However, our study concluded ZnO ENPs to be more toxic than CuO ones. This difference might be due to a different genetic make-up exhibited by different plants. Concerns are being raised by environmentalists and policy makers on the inevitable toxicological affects produced by ZnO and CuO ENPs in plant species [57,58]. So, there is scope for the conductance of further studies on these ENPs keeping in mind the organisms’ safety as they eventually become part of the food chain after getting released into the environment and cause nanotoxicity by disrupting their homeostatic mechanisms [59].

## 4. Materials and Methods

### 4.1. Synthesis and Characterization of ZnO and CuO ENPs

ZnO and CuO ENPs were synthesized by the coprecipitation method of Javed et al. [60,61] X-ray diffraction (XRD) and scanning electron microscopy (SEM) were performed using PANalytical Empyrean diffractometer and HITACHI S4800 (HITACHI, Ibraraki, Japan) respectively. Crystallinity was determined by XRD and SEM was carried out to elucidate the morphology of ENPs. Transmission electron microscopy (TEM) was performed using HITACHI H-7650 (HITACHI, Tokyo, Japan) to determine the size of ZnO and CuO ENPs. Dispersion stability of ENPs was determined through Zeta potential using Zeta sizer Nano ZS (Malvern, The Netherland).

### 4.2. Medium Preparation for Organogenesis

The Murashige and Skoog (MS) medium [62] and 3% (*w*/*v*) sucrose were used for the preparation of culture medium. Total 10 treatments were prepared, i.e., 5 containing 0, 2, 20, 200, and 2000 mg/L of ZnO ENPs, and 5 containing 0, 2, 20, 200, and 2000 mg/L CuO ENPs in MS culture medium for root organogenesis. pH was adjusted to 5.7–5.8 and 0.8% (*w*/*v*) plant agar was added at the end for solidification of media. Then, MS media was autoclaved at 1.06 kg cm^−2^ of pressure for 15 min at 121 °C.

### 4.3. Growth Conditions of Organogenesis

Candyleaf seeds were purchased from Shenyang Agricultural University (China). According to the [34] protocol, seeds were disinfected with 0.1% (*w*/*v*) of mercuric (II) chloride (HgCl_2_) and then cultured on plain MS medium. From 4 weeks old seedlings, axillary shoot nodes were removed and incubated in MS medium (without any plant growth regulators (PGRs)) for shoots formation. After another 4 weeks, the shoots obtained were shifted to different MS media treatments (without PGRs) containing different concentrations of ZnO and CuO ENPs in order to grow roots. A total of 15 shoots were used per treatment. The roots were grown after cultivation of shoots for 4 weeks in case of all treatments. The experiment was conducted in the growth room chamber in triplicate having a photoperiod of 16 h light and 8 h dark, provided with 24 ± 1 °C temperature, 55–60% rate of relative humidity, and cool-white fluorescent light having 35 μmol m^−1^s^−1^ irradiance. Later on, different parameters of growth, i.e., percentage (%) rooting of explants, mean length of regenerants and roots, mean number of nodes, leaves and roots, and mean fresh weight (FW) and mean dry weight (DW) of regenerants, stem and leaves were measured.

### 4.4. Steviol Glycosides (SGs) Analysis

According to the protocol of [34], leaves from regenerants were taken to extract SGs. The process involved washing of leaves, taken from different treatments, with sterile distilled water. Then, after filtering this material on filter paper, it was dried for 48 h in an oven at 60 °C.

The SGs of samples were analyzed using high performance liquid chromatography (HPLC) in accordance with the protocol of [34]. First of all, 20 mg of sample was added from each treatment to a microcentrifuge tube containing 1 mL of 70% (*v*/*v*) methanol. The samples were incubated for 15 min in ultrasonic bath at 55 °C. Later on, centrifugation was performed for 10 min at 12,000 rpm and 25 °C. The supernatant was collected and filtered by 0.22 µm PTFE Millipore syringe filters. At the end, all samples were run in triplicate for HPLC analysis using Ultimate 3000 (Thermo Fisher, Temecula, CA, USA.).

### 4.5. Antioxidant Assays

According to the protocol of [34] for extract preparation, the leaves of regenerants from different treatments were dried and 0.1 g of fine powder was taken. The powder was dissolved in 500 μL of dimethyl sulfoxide (DMSO). Afterwards, vortexing was performed for 5 min followed by 30 min sonication. This mixture was centrifuged at 10,000 rpm for 15 min. The supernatant was taken and stored at 4 °C until the performance of various antioxidant tests.

### 4.6. Quantification of Total Flavonoid Content (TFC)

TFC was determined from leaf extracts of Candyleaf according to the method of [34]. An aliquot of 20 µL (4 mg/mL) DMSO stock solution from each treatment was transferred to 96-well plate. Then, 10 µL of 1.0 M potassium acetate, 10 µL of 10% aluminum chloride, and 160 µL of distilled water was added to it. The mixture was kept for 30 min at room temperature. The absorbance of all samples that run in triplicate was calculated at 630 nm by a microplate reader. The standard used was quercetin and the results were expressed as quercetin in µg per mg (µg QE/mg).

### 4.7. Quantification of Total Phenolic Content (TPC)

TPC was determined from Candyleaf leaf extracts using the method of [34]. According to this procedure, an aliquot of 20 µL (4 mg/mL) of DMSO stock solution from each treatment was transferred to 96-well plate. Ninety microliters of the Folin–Ciocalteu reagent was added to it and the plate was kept for 5 min. Later on, 90 µL of sodium carbonate was added to this mixture. The samples that run in triplicate were used for calculating absorbance at 630 nm by a microplate reader. The standard taken was gallic acid and the results were expressed as gallic acid in µg per mg (µg GAE/mg).

### 4.8. Quantification of DPPH-FRSA

DPPH-FRSA was performed according to the protocol of [34]. It involved mixing of 10 µL (4 mg/mL) of leaf extracts with 190 µL of DPPH (0.004% *w*/*v* in methanol). This mixture was incubated for 1 h in darkness. The absorbance of all samples that run in triplicate was calculated at 515 nm by a microplate reader. The positive and negative controls were ascorbic acid and DMSO, respectively.

% inhibition of test sample = % scavenging activity = (1 − Abs/Abc) × 100

where, Abs specifies absorbance of sample with DPPH solution, and Abc indicates absorbance of DPPH solution only. Table curve software 2D Ver. 4 (SPSS Inc., Chicago, IL, USA) was used to calculate IC50.

### 4.9. Quantification of Total Antioxidant Capacity (TAC)

TAC was determined according to the procedure of [34]. One hundred microliters of aliquot was taken from the stock solution of each sample (4 mg/mL in DMSO). It was mixed with 900 µL of reagent solutions, i.e., 4 mM of ammonium molybdate, 28 mM of sodium phosphate, and 0.6 of M sulfuric acid. Then, this mixture was incubated for 90 min at 95 °C and cooled at room temperature. The absorbance of all samples that run in triplicate was calculated at 695 nm by a microplate reader. The standard used was ascorbic acid and the results were expressed as ascorbic acid µg per mg (µg AA/mg).

### 4.10. Quantification of the Total Reducing Power (TRP)

TRP of different treatment samples was calculated using the procedure of [34]. One hundred microliters of each sample (4 mg/mL in DMSO) was added to 96-well plate. Two hundred microliters of phosphate buffer (0.2 M, pH 6.6) and 250 µL of 1% (*w*/*v*) potassium ferricyanide were added to it. Then, incubation was carried out for 20 min at 50 °C. 200 µL of 10% (*w*/*v*) trichloroacetic acid was added to it. The mixture was centrifuged for 10 min at 3000 rpm. One hundred and fifty microliters of supernatant was taken and mixed with 50 µL of 0.1% (*w*/*v*) ferric chloride solution. The absorbance of all samples run in triplicate was calculated at 630 nm. The standard used was ascorbic acid and the results were expressed as ascorbic acid µg per mg (µg AA/mg).

### 4.11. Data Analysis

The experimental design was randomized and the data was statistically analyzed by SPSS, Version 17.0 (SPSS Inc., Chicago, IL, USA). ANOVA was used to calculate statistical difference, and the significance of difference between means ± SE (standard error) values was calculated by Duncan’s multiple range tests carried out at *p* < 0.05.

## 5. Conclusions

Summing up, this study described the modulating effect of ZnO and CuO ENPs employed to the MS culture medium of a medicinal plant, Candyleaf (*S. rebaudiana*). These ENPs significantly triggered and boosted the morphological and biochemical profiling of in vitro grown regenerants of Candyleaf up to a 2 mg/L dosage of ZnO ENPs and 20 mg/L of CuO ENPs concentration. However, our data illustrates that the alleviating impact of these ENPs was produced at 200 mg/L and 2000 mg/L of ZnO and CuO ENPs. This much higher dosage of these ENPs became toxic to the Candyleaf plant and is environment unfriendly. Nonetheless, our promising findings open ways to use positive aspects of metallic oxide ENPs for the enhanced production of bioactive metabolic ingredients of medicinal plants in in vitro batch cultures for the nutraceutical industry. Moreover, the detailed knowledge underlining the promotion of secondary metabolism via ENPs elicitation should be explored extensively in future research to overcome challenges in the health sector.

## Figures and Tables

**Table 1:** Zeta potential of ZnO and CuO ENPs.

Nanofluid	Zeta Potential (mV)	Conductivity (mS/cm)
ZnO	−12.8	0.0229
CuO	−11.7	0.0201

**Table 2:** Comparison of morphological parameters in 8 weeks old regenerants produced from shoots on the Murashige and Skoog (MS) medium supplemented with different concentrations of ZnO ENPs.

Conc. of ZnO NPs (mg/L)	% Rooting of Shoot Explants	Mean Length of Regenerants (cm)	Mean Length of Roots (cm)	Mean no. of Roots	Mean no. of Nodes	Mean no. of Leaves	FW of Regenerants (g)	FW of Leaves (g)
0	82.7	15.5 ± 0.68 ^c^	4.04 ± 0.68 ^b^	11.6 ± 0.68 ^b^	6.23 ± 0.02 ^b^	15.3 ± 0.26 ^c^	0.59 ± 0.03 ^c^	0.14 ± 0.02 ^c^
2	90.5	27.3 ± 0.66 ^a^	7.16 ± 0.92 ^a^	17.3 ± 1.76 ^a^	9.33 ± 0.33 ^a^	20.6 ± 0.66 ^a^	0.80 ± 0.16 ^a^	0.36 ± 0.06 ^a^
20 200	88.7 77.3	20.3 ± 1.52 ^b^ 12.3 ± 0.44 ^d^	2.83 ± 0.16 ^c^ 0.33 ± 0.16 ^d^	9.15 ± 2.30 ^c^ 2.33 ± 1.20 ^d^	5.66 ± 0.88 ^c^ 4.61 ± 0.88 ^d^	19.2 ± 1.76 ^b^ 10.2 ± 2.03 ^d^	0.72 ± 0.20 ^b^ 0.52 ± 0.07 ^d^	0.26 ± 0.06 ^b^ 0.09 ± 0.03 ^d^
2000	50.8	10.8 ± 0.26 ^e^	0.00 ± 0.11 ^e^	0.00 ± 0.01 ^e^	2.18 ± 0.01 ^e^	8.16 ± 0.12 ^e^	0.46 ± 0.05 ^e^	0.05 ± 0.01 ^d^

±: standard error, small alphabetical letters (a–e) with mean values representing the differences among treatments with in the columns according to Duncan’s multiple range test at a confidence level of 95%.

**Table 3:** Comparison of morphological parameters in 8 weeks old regenerants produced from shoots on MS medium supplemented with different concentrations of CuO ENPs.

Conc. of CuO NPs (mg/L)	% of Shoot Explants Rooting	Mean Length of Regenerants (cm)	Mean Length of Roots (cm)	Mean no. of Roots	Mean no. of Nodes	Mean no. of Leaves	FW of Regenerants (g)	FW of Leaves (g)
0	76.4	15.8 ± 0.51 ^c^	5.82 ± 0.35 ^c^	18.8 ± 1.91 ^c^	7.89 ± 0.54 ^d^	11.1 ± 1.63 ^c^	0.67 ± 0.07 ^b^	0.25 ± 0.01 ^b^
2	89.4	19.6 ± 0.66 ^b^	7.33 ± 0.60 ^b^	23.6 ± 1.45 ^b^	10.4 ± 0.33 ^b^	17.8 ± 0.02 ^b^	0.74 ± 0.17 ^a^	0.34 ± 0.02 ^a^
20 200	94.2 60.8	21.1 ± 1.16 ^a^ 12.6 ± 0.44 ^d^	8.54 ± 0.51 ^a^ 4.41 ± 0.08 ^d^	26.3 ± 0.88 ^a^ 6.02 ± 1.15 ^d^	11.0 ± 1.15 ^a^ 8.33 ± 0.88 ^c^	19.3 ± 1.76 ^a^ 7.12 ± 2.09 ^d^	0.76 ± 0.13 ^a^ 0.62 ± 0.04 ^c^	0.36 ± 0.03 ^a^ 0.13 ± 0.02 ^c^
2000	48.5	10.4 ± 0.21 ^e^	0.00 ± 0.02 ^e^	0.00 ± 0.08 ^e^	3.02 ± 0.21 ^e^	5.16 ± 0.37 ^e^	0.55 ± 0.02 ^d^	0.08 ± 0.01 ^d^

±: standard error, small alphabetical letters (a–e) with mean values representing the differences among treatments with in the columns according to Duncan’s multiple range test at a confidence level of 95%.

## Abbreviations

MS: Murashige and Skoog; HPLC, high performance liquid chromatography; TFC, total flavonoid content; TPC, total phenolic content; DPPH, 2,2-diphenyl-1-picryl hydrazyl; TAC, total antioxidant capacity; TRP, total reducing power; DMSO, dimethyl sulphoxide; ROS, reactive oxygen species.
